# Association of soluble endothelial protein C receptor plasma levels and *PROCR* rs867186 with cardiovascular risk factors and cardiovascular events in coronary artery disease patients: The Athero *Gene* Study

**DOI:** 10.1186/1471-2350-13-103

**Published:** 2012-11-08

**Authors:** Choumous Kallel, William Cohen, Noémie Saut, Stefan Blankenberg, Renate Schnabel, Hans J Rupprecht, Christoph Bickel, Thomas Munzel, David-Alexandre Tregouet, Pierre-Emmanuel Morange

**Affiliations:** 1 INSERM UMR_1062, Aix-Marseille Université, Marseille, F-13385, France; 2 Department of General and Interventional Cardiology, University Heart Center Hamburg, Hamburg, Germany; 3 Department of Medicine II, Johannes Gutenberg-University Mainz, Mainz, Germany; 4 Federal Armed Forces Central Hospital Koblenz, Koblenz, Germany; 5 INSERM, UMR_S 937; Institute of Cardiometabolism And Nutrition (ICAN), Université Pierre et Marie Curie Paris 6, Paris, F-75013, France

**Keywords:** \, Haemostasis, Protein C, Endothelial protein C receptor, Coronary artery disease

## Abstract

**Background:**

Blood coagulation is an essential determinant of coronary artery disease (CAD). Soluble Endothelial Protein C Receptor (sEPCR) may be a biomarker of a hypercoagulable state. We prospectively investigated the relationship between plasma sEPCR levels and the risk of cardiovascular events (CVE).

**Methods:**

We measured baseline sEPCR levels in 1673 individuals with CAD (521 with acute coronary syndrome [ACS] and 1152 with stable angina pectoris [SAP]) from the Athero*Gene* cohort. During a median follow up of 3.7 years, 136 individuals had a CVE. In addition, 891 of these CAD patients were genotyped for the *PROCR* rs867186 (Ser219Gly) variant.

**Results:**

At baseline, sEPCR levels were similar in individuals with ACS and SAP (median: 111 vs. 115 ng/mL respectively; p=0.20). Increased sEPCR levels were found to be associated with several cardiovascular risk factors including gender (p=0.006), soluble Tissue Factor levels (p=0.0001), diabetes (p=0.0005), and factors reflecting impaired renal function such as creatinine and cystatin C (p<0.0001). sEPCR levels were not significantly associated with the risk of CVE (median: 110 and 114 ng/mL in individuals with and without future CVE respectively; p=0.68). The rs867186 variant was found to explain 59% of sEPCR levels variability (p<10^-200^) but did not associate with CVE risk.

**Conclusion:**

Our findings show that in patients with CAD, circulating sEPCR levels are related to classical cardiovascular risk factors and renal impairment but are not related to long-term incidence of CVE.

## Background

Coronary artery disease (CAD) is the leading cause of death in the developed world [1]. It is an inflammatory process that involves cellular and molecular responses to endothelial dysfunction [2]. One such response is blood coagulation, and recent studies demonstrate that blood coagulation is an essential determinant of the risk of CAD complications [2,3].

The protein C (PC) anticoagulant pathway plays a pivotal role in controlling thrombosis and in limiting the inflammatory response. It may also reduce endothelial cell apoptosis in response to inflammatory cytokines and ischemia [3,4]. The endothelial PC receptor (EPCR) is important to these processes. Mainly expressed on the endothelial cells of large vessels [5-7], by binding to PC, EPCR accelerates the rate of PC activation approximately twenty fold *in vivo*[8]. Once PC is activated (Activated PC, APC), EPCR also mediates its anti-apoptotic effect on endothelial cells [9].

In addition to endothelial cell-bound EPCR, a soluble form of EPCR (sEPCR) circulates in human plasma resulting from EPCR membrane shedding mediated by a metalloprotease [7,10], probably TACE/ADAM17 [11]. This process occurs constitutively and is amplified by thrombin and some inflammatory cytokines (e.g., TNFα, IL-1β) [4,11]. sEPCR binds PC and APC with the same affinity as the original membrane form of EPCR and may inhibit both PC activation and APC anticoagulant activity [12]. In addition, sEPCR modulates inflammation by binding to activated neutrophils [13,14] and also reportedly binds to factor VIIa, reducing the ability of FVIIa to activate FX [15]. Moreover, high levels of plasma sEPCR were observed in patients with clinical conditions in which thrombin is generated, such as CAD [13,16], and decreased sEPCR levels were observed in another study of patients on anticoagulant therapy [14].

These data all suggest that sEPCR may act as a procoagulant by reducing antithrombotic and anti inflammatory effects. However, data regarding the association between sEPCR plasma levels and the risk of thrombosis are sparse and contradictory. Uitte de Willige et al.[17], reported that a high level of sEPCR increased the risk of venous thrombosis, whereas another retrospective case/control study showed that patients with increased sEPCR levels had a reduced risk of myocardial infarction (MI) [18].

Several studies demonstrated that sEPCR levels were strongly genetically controlled [17-22]. The rs867186 diallelic single nucleotide polymorphism in the *PROCR* gene (g.6936A_G, c.4600A_G), resulting in a serine-to glycine substitution at codon 219 in the membrane-spanning domain of EPCR, explains between 56% and 87% of the variations in sEPCR levels [17,20,23]. The G allele tags the A3 haplotype (4 common *PROCR* haplotypes have been identified in whites) and is associated with increased shedding of EPCR from the endothelial membrane, both by rendering the receptor more sensitive to cleavage [24] and by leading to a truncated mRNA through alternative splicing [25]. Besides this important genetic effect, little is known about the association between sEPCR plasma levels and other environmental cardiovascular risk factors.

Since markers of procoagulable state are of major relevance to CAD, sEPCR could be a risk factor or a predictor of cardiovascular events (CVE) in individuals with CAD.We tested this hypothesis in the Athero*Gene* prospective cohort. We also studied the relation between sEPCR levels and conventional cardiovascular risk factors.

## Methods

### Study population

The Athero *Gene* study is a prospective cohort of CAD patients enrolled during several successive phases of recruitment between November 1996 and February 2004 [26]. Briefly, patients who underwent coronary angiography at the Medical Department of the Johannes Gutenberg-University Mainz or the Bundeswehrzentralkrankenhaus Koblenz and who had at least one stenosis >30% diagnosed in a major coronary artery were enrolled in the cohort. Unstable angina was classified by Braunwald classification (class B or C). Follow-up information was obtained on non-fatal myocardial infarction (MI) and on death from cardiovascular (CV) causes (fatal MI, heart failure as a consequence of MI, ventricular arrhythmia, fatal stroke and other cause of vascular deaths). Information on the cause of death was obtained from the hospital or from the patient’s general practitioner.

Among patients recruited in the early phase of the study, insufficient plasma remained for sEPCR testing. Therefore, this study included only patients recruited after June 1999 (n = 1673 - second round of the Athero*Gene* Study). Among these, 525 (31%) presented an acute coronary syndrome (ACS) at entry (314 unstable angina and 211 acute MI). The remaining individuals presented a stable angina pectoris (SAP) at entry. All individuals were followed up for a median time of 3.7 years (maximum 6.2) and 136 experienced a CVE (71 non-fatal MI and 65 CV deaths).

Study participants had German nationality, were inhabitants of the Rhein-Main area, and were of European descent. The study was approved by the ethics committee of the University of Mainz. Participation was voluntary, and each participant gave written informed consent.

### Laboratory methods

Blood was drawn from all study subjects under standardized conditions before coronary angiography was performed. Samples were stored at −80°C until analysis. Plasma sEPCR levels were measured by enzyme linked ImmunoSorbent Assay (ELISA) according to the manufacturer’s instructions. The asserachrom sEPCR ELISA kits were from Diagnostica Stago (Asnière, France) and the inter-assay variability was 7.5%. Other biological parameters were measured as previously described [27].

### Genotype analysis

DNA was available in a subsample of 891 CAD patients among which 77 experienced a CVE during the follow-up. In these patients, five *PROCR* single nucleotide polymorphisms (SNPs), including the *PROCR* rs867186 (Ser219Gly), were typed using the Affymetrix Genome-Wide Human SNP 6.0 array as part of a previously described genome-wide association study [28].

### Statistical analysis

Associations between baseline cardiovascular risk factors and CVE were tested by ANOVA and Chi^2^ analyses. Associations between sEPCR levels, haemostatic parameters and other cardiovascular risk factors were investigated through Pearson correlation coefficients adjusted for age and sex. sEPCR was log transformed to remove positive skewness. The relationship between sEPCR (considered as continuous variables or interquartiles) and CVE was tested by Cox regression analysis. Two models were successively fitted: model 1was first adjusted for age and sex; model 2 was additionally adjusted for clinical status (ACS vs. stable angina), smoking status, body mass index, diabetes, hypertension, HDL-cholesterol, triglyceride, CRP, number of stenosed vessels, and medication use (heparin, beta-blockers, ACE-inhibitors, calcium antagonists and statins).

Association of *PROCR* SNPs with CVE was tested by the Cochran-Armitage trend test [29] and by a Cox regression analysis, while their association with log sEPCR levels was tested by a linear model. Linkage disequilibrium and haplotype analyses of *PROCR* SNPs were conducted using the THESIAS software [30].

All analyses were performed with SAS software, version 9.1 (SAS Institute Inc., Cary,NC, USA). P-values< 0.05 were considered statistically significant.

## Results

### Baseline characteristics of individuals according to cardiovascular outcome

Table 1 shows the baseline characteristics of the CAD patients according to outcome. In the group with occurrence of CVE during follow-up, there was a higher proportion of females, patients presented more often with an ACS and with a history of previous MI, and a higher prevalence of diabetes was observed. The number of stenosed coronary arteries was also higher in this group. There was a marked increase in CRP, TAFIa/TAFIai and TFPI levels, and a slight elevation in D-dimer levels. Factors reflecting deteriorationof renal function such as creatinin and cystatin C were also markedly increased.

**Table 1:** Baseline characteristics of coronary artery disease (CAD) patients according to the outcome during follow-up

**Characteristics**	**No cardiovascular event n=1537**	**Cardiovascular event n=136**	**Association Test**
Age, years	61.2 ± 9.5	62.8 ± 10.5	p = 0.059
Females	323 (21 %)	39 (29%)	p = 0.050
Acute coronary syndrome	467 (30 %)	58 (43 %)	p = 3.78 10^-3^
Previous myocardial infarction	586 (38 %)	65 (48 %)	p = 0.028
Number of stenosed coronary arteries			
one vessel	438 (29 %)	21 (15 %)	
two vessels	479 (31 %)	43 (32 %)	p = 1.31 10^-3^
three vessels	620 (40 %)	72 (53 %)	
Body mass index (kg/m^2^)	27.7 ± 3.9	27.8 ± 3.8	p = 0.927
Current smoker	304 (20 %)	33 (24 %)	p = 0.219
Diabetes mellitus	240 (16 %)	43 (32 %)	p = 1.17 10^-5^
Hypertension	1151 (75 %)	105 (77 %)	p = 0.606
Medications at enrollment			
Heparin	521 (34 %)	55 (40 %)	p = 0.132
Antiplatelet therapy	1327 (86 %)	108 (79 %)	p = 0.039
Statins	800 (52 %)	68 (50 %)	p = 0.277
Beta-blocker	1026 (67 %)	80 (59 %)	p = 0.072
ACE-inhibitor	770 (50 %)	84 (62 %)	p = 9.41 10^-3^
Calcium antagonists	197 (13 %)	25 (18 %)	p = 0.085
Total cholesterol (mgdL^-1^)	197 ± 45	205 ± 51	p = 0.038
HDL-cholesterol (mgdL^-1^)	49.4 ± 13.5	48.1 ± 13.6	p = 0.290
Triglyceride (mgdL^-1^)	129 (95–182)	130 (100–183)	p = 0.143
CRP (mgL^-1^)	2.39 (1.02 - 6.14)	4.59 (1.89 - 11.7)	p = 8.41 10^-6^
Fibrin monomers (μmL)	3.90 (2.64 - 5.33)	3.32 (2.64 - 5.34)	p = 0.514
D-dimers (μgmL^-1^)	0.34 (0.24 - 0.52)	0.39 (0.25 - 0.78)	p = 0.024
t-TAFI (μg mL^-1^)	12.0 ± 2.7	12.4 ± 2.7	p = 0.196
TAFIa/TAFIai (ngmL^-1^)	10.48 (8.13 - 13.95)	11.59 (9.00 - 15.08)	p = 2.75 10^-3^
Soluble Tissue factor (pgmL^-1^)	158 (124–204)	159 (124–208)	p = 0.641
f-TFPI (ngmL^-1^)	10.80 (7.61 - 18.89)	13.46 (9.34 - 25.89)	p = 7.54 10^-3^
Creatinine (mgdL^-1^)	0.96 ± 1.03	1.03 ± 0.29	p = 5.59 10^-4^
Cystatin C (mgL^-1^)	0.81 (0.71 - 0.94)	0.86 (0.72 - 1.08)	p = 1.28 10^-4^
sEPCR(ngmL^-1^)	114 (93–161)	110 (91–177)	p = 0.654

t-TAFI measures total Thrombin Activating Fibrinolysis Inhibitor in plasma ; TAFIa/TAFIai measures activated TAFI levels in plasma; f-TFPI = free form of tissue factor pathway inhibitor 1.

Categorical variables are presented as n (%), and continuous variables as mean ± SD or median (25^th^ - 75^th^ percentile) for skewed variables (for these variables, tests were performed on log-transformed distribution).

### Association between sEPCR levels and cardiovascular risk factors

sEPCR levels were similar in patients with ACS at baseline compared to those with SAP at baseline (p=0.20; Table 2). The highest correlations between sEPCR and other biological measurements were observed for creatinin (r = 0.14) and cystatin C (r = 0.17) (Table 2). Other significant correlations were found with soluble tissue factor (sTF) (r = 0.11) and total amount of TAFI (t-TAFI) (r = 0.08). Plasma sEPCR levels were higher in males and in diabetic patients, but were decreased in smokers.

**Table 2:** Association between sEPCR, haemostatic parameters and other cardiovascular risk factors, adjusted for age and sex

	**sEPCR**	
	**Pearson’s partial correlation coefficients**	p-value*^1^
Age	0.03	p=0.27
Body mass index	0.05	p=0.06
Total cholesterol	−0.02	p=0.40
HDL cholesterol	−0.02	p=0.31
Triglyceride	0.05	p=0.04
CRP	−0.06	p=0.01
Fibrin monomer	0.02	p=0.48
D-dimers	0.00	p=0.89
t-TAFI	0.08	p=0.0007
TAFIa/TAFIai	0.03	p=0.19
Soluble Tissue factor	0.11	p<0.0001
f-TFPI	−0.02	p=0.38
Creatinine	0.14	p<0.0001
Cystatine C	0.17	p<0.0001
	**Median (interquartile range)**	p-value*^2^
Sex		
Male	116 (94–166)	
Female	107 (89–155)	p=0.006
Acute coronary syndrome		
No	115 (94–166)	
Yes	111 (90–156)	p=0.20
Current smoking		
No	115 (94–170)	
Yes	108 (88–148)	p=0.005
Diabetes		
No	111 (91–158)	
Yes	123 (101–182)	p=0.0005
Hypertension		
No	111 (91–147)	
Yes	115 (94–168)	p=0.12

* Tests performed on correlations (^1^) or means (^2^) adjusted on age and sex; skewed variables were log-transformed.

### Association between sEPCR levels and cardiovascular outcome

sEPCR levels were not significantly different between individuals with or without future CVE, regardless of whether sEPCR was studied as continuous variable or in quartiles (Table 3). Likewise, there was no significant association when individuals with ACS at baseline were studied separately from those with SAP at baseline (data not shown).

**Table 3:** Hazard ratios (95% confidence interval) for cardiovascular death or myocardial infarction according to quartiles of baseline sEPCR levels

	**Q1**	**Q2**	**Q3**	**Q4**	**p (continuous scale)**
Quartiles ranges	48 - 93	94 - 113	114 - 162	163 - 600	
Patients with events/ all patients	36/418	35/418	29/419	36/418	
Model 1	reference	1.00 (0.63 - 1.60)	0.85 (0.52 - 1.39)	1.03 (0.65 - 1.64)	p=0.57
		p=0.99	p=0.51	p=0.89	
Model 2	reference	1.11 (0.67 - 1.83)	0.96 (0.57 - 1.62)	1.13 (0.69 - 1.87)	p=0.38
		p=0.69	p=0.87	p=0.63	

Model 1: adjusted on age and sex; model 2: adjusted on age, sex, acute coronary syndrome, smoking status, body mass index, diabetes, hypertension, HDL-cholesterol, triglyceride, CRP, number of stenosed vessels, and medication use (heparin, beta-blockers, ACE-inhibitors, calcium antagonists and statins). P-value was calculated on continuous log-transformed sEPCR.

### PROCR SNPs analysis

In the subsample of CAD patients with DNA available, five *PROCR* SNPs were genotyped: rs6088738, rs6088747, rs2069940, rs867186 and rs1415774. These SNPs were in strong linkage disequilibrium, with rs6088747 and rs867186 being in complete association (pairwise r^2^~1) with rs1415774 and rs2069940, respectively (Table 4). None of these SNPs were associated with the risk of future CVE (Table 5). In particular, the allele frequency of the rs867186-G allele was 0.11 both in patients with and without future CVE (Table 5). As expected, the rs867186 variant was strongly associated with sEPCR levels and explained 59.1% (p < 10^-200^) of their variability. The rs867186-G allele was associated with increased sEPCR levels in a fairly additive fashion, the observed log sEPCR levels being 4.66±0.29, 5.48±0.26 and 5.90±0.37 in AA (n = 708), AG (n = 172) and GG (n = 11) carriers. This association was homogeneous in patients with or without future CVE, in men and in women, and was not modified after adjusting for the studied cardiovascular risk factors (data not shown). Further haplotype analysis revealed that the rs867186-G allele was carried out by a single haplotype, that was the only one associated with sEPCR levels (Table 6).

**Table 4:** Pairwise linkage disequilibrium observed at the PROCR locus in the AtheroGene study (n = 891)

	**rs6088738**	**rs6088747**	**rs2069940**	**rs867186**	**rs1415774**
rs6088738	-	−0.97	−0.93	−0.93	−1
rs6088747	0.21	-	−0.98	−0.98	0.99
rs2069940	0.03	0.09	-	1	−1
rs867186	0.03	0.09	1	-	−1
rs1415774	0.21	0.97	0.09	0.09	1

Pairwise linkage disequilibium was expressed in terms of D' (upper-right triangle) and r^2^ (bottom-left triangle) values.

**Table 5:** **Genotype distribution of the*PROCR*polymorphisms in CAD patients according to the outcome during follow-up**

	**No cardiovascular event N = 805**	**Cardiovascular events N = 77**
	rs6088738	
GG	469 (59%)	46 (60%)
GA	291 (36%)	28 (36%)
AA	40 (5%)	3 (4%)
MAF^(1)^	0.232	0.221
P^(2)^	p = 0.752	
	rs6088747	
TT	280 (35%)	18 (24%)
TG	380 (47%)	43 (56%)
GG	145 (18%)	16 (20%
MAF	0.416	0.487
P	p = 0.092	
	rs867186	
AA	639 (79%)	62 (80%)
AG	157 (20%)	13 (17%)
GG	9 (1%)	2 (3%)
MAF	0.109	0.110
P	p = 0.948	

^(1)^ MAF: Minor Allele Frequency.

^(2)^ P-value of the Cochran-Armitage trend.

As rs2069940 and rs1415774 were in complete association with rs867186 and rs6088747, respectively, their genotype distributions were not displayed.

**Table 6:** **Association of the main*PROCR*haplotypes with sEPCR (log) levels in the AtheroGene study (n = 891)**

**Polymorphisms**					**Haplotype Frequencies**	Haplotypic Effects^*^
**rs6088738**	**rs6088747**	**rs2069940**	**rs867186**	**rs1415774**		
G	T	G	G	G	0.108	+0.786 [0.742 - 0.829] p = 6.74 10^-270^
G	T	C	A	G	0.239	−0.027 [−0.061 - 0.007] p = 0.125
G	G	C	A	A	0.416	reference
A	T	C	A	G	0.228	0.028 [−0.009 - 0.066]p = 0.143
Global test for haplotypic association					χ^2^ with 3 df = 792.6 p =1.71 10^-171^	

* Additive effects of each inferred haplotype compared to the most frequent one used as the reference haplotype. Effects were adjusted for age, sex, smoking and CV events.

Of note, in this sample, the Hazard Ratio (HR) for future CVE associated with an increase of log-sEPCR (on continuous scale) was 0.84 [0.35 - 2.02] (p = 0.69) in carriers of the rs867186 AA genotype while an opposite trend (HR of 4.88 [0.87 - 27.4] (p = 0.07) was observed in carriers of the rs867186-G allele. The test for homogeneity of these two HRs was borderline (p = 0.075).

## Discussion

To the best of our knowledge, this is the first prospective study that investigates the association between sEPCR levels and CAD. Contrary to our initial hypothesis, there was no association between sEPCR levels and future CVE. Moreover both individuals with ACS and SAP at baseline had similar sEPCR levels.

Only one previously published case–control study examined the relationship between sEPCR levels and CAD [18]. In this work, stratification of sEPCR in quartiles according to the levels in controls showed that, compared to the first quartile, the OR for subjects with values in the 4^th^ quartile was 0.57 (95CI: 0.34-0.95). This result differs from ours, as we did not find a protective effect of high sEPCR levels in our cohort. Among the possible explanations for this discrepancy are differences in study design (retrospective versus prospective) and in the age of study participants at baseline (median age of 42 years in the case–control study versus 62 years in our prospective study). It could also be argued that the low number of events observed during the follow-up with median time of 3.7 years may have limited our power to detect any association of sEPCR with future CVE, especially if sECPR effects, if any, exert at a later time period. Nevertheless, our study was large enough to detect the association of several biomarkers, including parameters characterizing the renal function, with the risk of future CVE.

The physiological role of sEPCR is still unclear. Elevated plasma sEPCR levels may increase thrombotic risk by inhibiting PC and APC and by competing with membrane associated EPCR for PC binding [12]. High plasma sEPCR levels might also result in low residual EPCR levels on the membrane, resulting in reduced PC activation. Alternatively, higher levels of endothelial or soluble EPCR may shift the haemostatic balance toward anticoagulant activity by inhibiting the activation of FX by the FVIIa-tissue factor (TF) complex. Low sEPCR levels, on the other hand, also might reflect increased thrombotic risk. This could be caused by low EPCR expression on the endothelium or by membrane-bound EPCR that is resistant to ADAM17 shedding [24], resulting in decreased APC formation. Further studies are needed to investigate the relationship between EPCR membrane expression and its circulating form. Indeed, a recent study reported that TNFα causes a rapid down-regulation of membrane associated EPCR expression without markedly affecting the spontaneous release of sEPCR by arterial endothelial cells [31].

With respect to parameters affecting sEPCR levels, we confirmed in a subsample of 891 patients who had both sEPCR measured and DNA available the major impact of the Ser219Gly *EPCR* polymorphism on sEPCR levels [17-22]. However, we did not observe any evidence in favour of an association of Ser219Gly with future CVE. This is unlikely due to a loss power since the same allele frequencies were observed in both groups of patients with or without future CVE. Conversely, this is in line with a recent review demonstrating that this polymorphism is unlikely a risk variant for arterial thrombosis but more likely a risk variant for venous thrombosis [32]. Nevertheless, it would be highly interesting to investigate whether the trend of association observed between sEPCR and CVE risk in rs867186-G carriers only could replicate in a much larger cohort with a longer follow-up.

In addition, we have explored the association of plasma sEPCR levels with haemostatic variables. sEPCR levels correlated with sTF and t-TAFI levels. Previous studies demonstrated that sTF levels, but not t-TAFI, were predictive of cardiovascular death in individuals with CAD [27,33]. In atherosclerosis, circulating sTF can arise not only by membrane shedding but also by alternative splicing [34]. Several recent observations indicate that FVIIa interacts with EPCR *in vivo*[35]. Moreover, analysis of FVII, FVIIa, and sEPCR levels in a large group of healthy individuals revealed that those with higher sEPCR levels also had higher levels of circulating FVII and FVIIa [19]. The association observed between sEPCR and sTF in the present study underlines the interplay between EPCR and the extrinsic coagulation pathway.

Several papers [14,36] have previously suggested that sEPCR levels could be a reliable marker of thrombin generation. Our study did not favour this hypothesis as no relation was observed between sEPCR levels and markers of thrombin generation such as D-dimer, fibrin monomers, and TAFIa/TAFIai levels.

We also evaluated the relationship between sEPCR levels and traditional cardiovascular risk factors. We confirmed recent data [31] demonstrating that gender strongly correlates with sEPCR levels, with higher circulating sEPCR levels observed in males. We found a strong association between diabetes and sEPCR levels. These results are in line with those from Ireland et al. [23] who observed a contribution of duration of diabetes on sEPCR levels in the Ealing Diabetes Study of Coagulation (EDSC). Interestingly, we also found a strong correlation between sEPCR and parameters reflecting kidney functions such as creatinine and cystatin C. High sEPCR levels were reported in hemodialysis patients and significantly decreased after kidney transplantation [37]. This finding extends those already reported on the association between coagulation parameters and kidney function [38].

## Conclusion

In conclusion, we reported the first prospective study investigating the association of sEPCR with CAD. We observed no association between sEPCR levels and acute coronary syndrome or with future cardiovascular events. However, sEPCR levels were associated with conventional cardiovascular risk factors such as diabetes and parameters reflecting kidney function. More research is warranted to elucidate the pathogenic effect of sEPCR in CAD.

## Competing interests

The authors declare that they have no competing interests.

## Authors' contributions

SB, HJR, CB, TM, DAT and PEM contributed to the design of this study. NS, SB, RS, HJR, CB and TM contributed to data acquisition. CK and WC participated to the statistical analysis of the data under the supervision of DAT. RS, DAT and PEM wrote the manuscript. All authors read and approved the final manuscript.

## Pre-publication history

The pre-publication history for this paper can be accessed here:

http://www.biomedcentral.com/1471-2350/13/103/prepub
